# Telehealth and Remote Interventions for Children With Cerebral Palsy: Scoping Review

**DOI:** 10.2196/36842

**Published:** 2022-10-17

**Authors:** Marina Pagaki-Skaliora, Eileen Morrow, Tim Theologis

**Affiliations:** 1 Nuffield Department of Orthopaedics, Rheumatology and Musculoskeletal Sciences University of Oxford Oxford United Kingdom; 2 Oxford University Hospitals National Health Service Foundation Trust Oxford United Kingdom

**Keywords:** cerebral palsy, CP, assistive technology, scoping review, software, application, telehealth, telerehabilitation, rehabilitation, COVID-19, children, health intervention, health care, digital intervention

## Abstract

**Background:**

Remote treatment, or telehealth, has shown promise for children with cerebral palsy (CP) prior to 2020; however, the beginning of the global COVID-19 pandemic limiting access to hospitals for face-to-face treatments has driven the need for telehealth and led to a surge in its development. Due to the recent developments, there has been limited synthesis of the available evidence of telehealth for children with CP.

**Objective:**

This study aimed to analyze and summarize the existing evidence for telehealth interventions for the treatment of children with CP and identify any areas requiring further research.

**Methods:**

A scoping review was performed. A systematic search of available literature in MEDLINE and PubMed was performed during July 2021. Inclusion criteria for articles were primary research and systematic reviews that investigated telehealth, included children with CP, were published between 2010-2021, and were written in English. Exclusion criteria were secondary research other than systematic reviews; interventions that did not meet the World Health Organization definition of telehealth; or studies where all participants were aged >18 years, children’s results were not reported separately, or there were no results reported for children with CP. A scoping review was chosen due to the expected heterogeneity of the participants, as well as the expected small sample sizes and inconsistency of measured outcomes; therefore, a narrative reporting of the results was considered appropriate.

**Results:**

In all, 5 papers were identified, which included the results of 11 studies—2 of the included articles were systematic reviews, which included the results of 3 studies each. These 6 studies, together with 5 primary research articles, were included in this scoping review. The existing evidence is of low methodological quality, primarily consisting of case series. There is some evidence that the requirements of telehealth differ depending on the children’s developmental stage and functional level. Telehealth is reported to reduce caregiver burden. There is mixed evidence on children’s compliance with telehealth. Overall, the results of telehealth interventions for the treatment of children with CP were positive, indicating either comparable or improved results compared with children receiving usual face-to-face care.

**Conclusions:**

The evidence base is lacking in breadth and methodological quality to provide robust clinical recommendations. Most studies investigated hand function only, indicating the limited scope of existing research. However, this review shows that telehealth has demonstrated potential to improve function for children with CP while making health care services more accessible and reducing caregiver burden. Areas requiring further research include telehealth interventions for the lower limb, postural management, and pain control and the barriers to implementing telehealth.

## Introduction

### Background

Cerebral palsy (CP) is an umbrella term for neurological and movement disorders caused by nonprogressive disturbance to the developing fetal or infant brain [1,2].

CP affects approximately 1 in 400 children in the United Kingdom and represents a lifetime disability with substantial socioeconomic consequences [2]. Functional mobility is best classified by the Gross Motor Function Classification System (GMFCS) [3], an international standard based on the severity of motor disability. GMFCS has been produced to allocate patients aged 6-18 years to 1 of 5 functional levels from “walking without limitations” (GMFCS level I) to “transported in a manual wheelchair” (GMFCS level V), which assist in defining the management plan. This system can be used by all health care professionals in the multidisciplinary team including dietitians, doctors, occupational therapists, orthotists, physiotherapists, psychologists, and speech and language therapists [4]. CP is also classified according to affected body areas as unilateral (hemiplegia) or bilateral (diplegia or quadriplegia, affecting predominantly lower limbs or all 4 limbs, respectively).

CP can be further classified by neurological pattern as dystonic, dyskinetic, ataxic, and mixed [3]. In 70% of cases, CP predominantly causes spasticity—increased muscle tone or tightness due to prolonged contraction. The increased muscle tone leads to progressive stiffness and deficient longitudinal muscle growth, which, in turn, causes secondary joint contracture, bone deformity, and pain. Dyskinetic CP is an extrapyramidal type and results from damage in the basal ganglia (BG). This area of the brain is damaged in Parkinson disease and as such, CP lesions in the BG can have a Parkinsonian presentation. Dystonic CP mostly occurs later in the antenatal period at 38-40 weeks of gestation, where metabolic demands of the BG area in the fetal brain lead to movement disorders such as dystonia. The BG can also be damaged due to hyperbilirubinemia as by-products from bilirubin metabolism are deposited in the brain and lead to dyskinesia. Children with dystonic CP present with dystonia and chorea [2,5]. Ataxic CP is another extrapyramidal type causing balance issues. The damage is in the involuntary motor neurons that affect coordination and gait. Children with ataxic CP often present with hypotonia, intention tremor, nystagmus, trunk ataxia, and balance problems [2].

### Treatment

CP affects many aspects of a person’s activities of daily living including movement and posture, speech and communication, and swallowing (eating and drinking) and causes a range of health problems such as osteopenia, excess saliva, pain and discomfort, stress and anxiety, depression, and sleep disturbances. Therefore, the treatment options and requirements are vast.

Modern clinical management has evolved to allow clinicians a greater variety of treatment options and offers patients more tailored treatment for their needs. Telehealth (TH) and remote interventions to facilitate the use of TH are at the forefront of this evolution. The World Health Organization (WHO) defines TH as “the delivery of healthcare services, where patients and providers are separated by distance. TH uses information and communication technologies for the exchange of information for the diagnosis and treatment of diseases and injuries, research and evaluation, and for the continuing education of health.” [6].

TH has shown promise for patients with CP prior to 2020 [7,8]. However, the beginning of the global COVID-19 pandemic limiting access to hospitals for face-to-face treatments has driven the need for remote treatments including TH and led to a surge in its development [9].

### Study Aims

The aim of this scoping review was to analyze and summarize the existing evidence for TH interventions for the treatment of children with CP and identify any areas requiring further research.

This review is timely due to the ongoing COVID-19 pandemic increasing the need for and development of TH interventions. This study will contribute to the current evidence base as despite 2 previous systematic reviews having been conducted in this area, neither was specific to children with CP, and 1 focused only on therapy for the upper extremity [10,11].

## Methods

### Study Design

**Figure 1:**
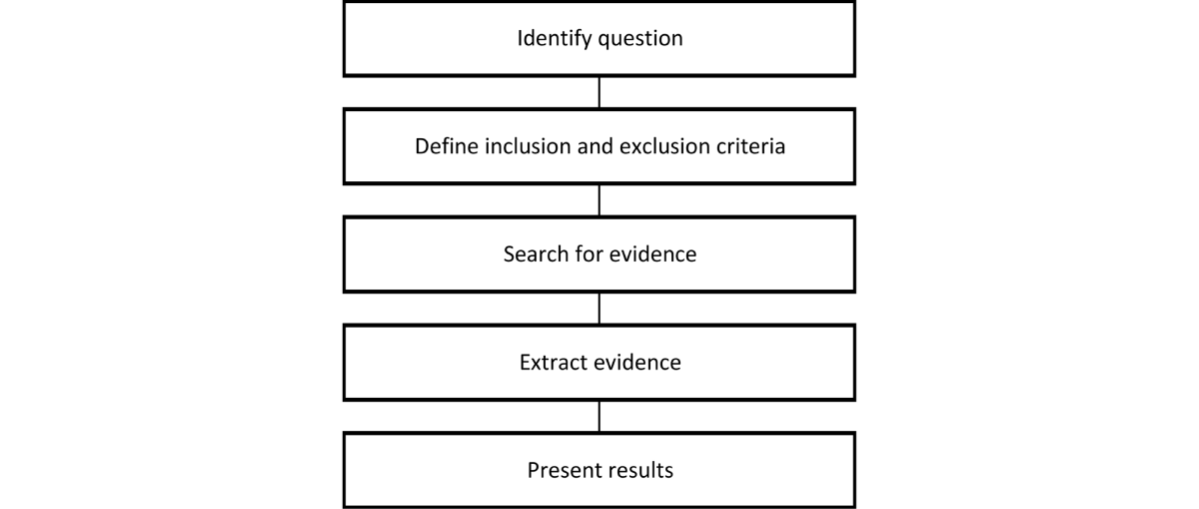
Scoping review methodology flowchart.

A scoping review was chosen as the appropriate methodology, as the substantial heterogeneity of the participants, small sample sizes, and an inconsistency of measured outcomes were expected. Therefore, narrative synthesis and reporting were considered the most appropriate. A scoping review also allowed a broad research question to highlight gaps in the literature and provide recommendations for TH as a method to assist children with CP in their ongoing treatment. The methods for the scoping review are summarized in Figure 1. The steps of a scoping review were followed and the PRISMA-ScR (Preferred Reporting Items for Systematic Reviews and Meta-Analyses Extension for Scoping Reviews) checklist completed (see Multimedia Appendix 1 [12]).

### Search Strategy

For the purpose of this paper, we used the definition of TH described by the WHO [6] as discussed in the introduction. Children were defined as aged 0-18 years.

A database search was conducted in the MEDLINE and PubMed databases. The MEDLINE search was completed using the OvidSP search platform, and the website-specific search engine was used to search the PubMed database. The search strategy used in the MEDLINE database is included in Table 1.

The International Journal of Rehabilitation Research was also manually searched for further articles that met the eligibility criteria, as it was identified a priori as having published several important papers in TH for people with CP. Backward reference searching was also conducted on all papers identified for inclusion through the previous search strategies. Authors were not contacted for further data.

The management of children with CP is multidisciplinary and includes a variety of aspects of their complex disability. For the purpose of this study, we did not limit the definition of “treatment” to 1 or more of the disciplines but rather considered children’s management globally, including all relevant disciplines involved in their care.

**Table 1:** The database search strategy with the number of articles for the MEDLINE database.

Number	Search term	Article, n
1	Cerebral palsy	23,791
2	Assistive tech*	2431
3	Treatment*	4,925,573
4	Software*	197,274
5	Application*	1,365,926
6	2 OR 4 OR 5	1,538,901
7	1 AND 3 AND 6	231

### Eligibility Criteria

Inclusion criteria for articles were primary research, systematic reviews, and meta-analyses that investigated TH, included children with CP either as all participants or results reported as a subgroup, were published between 2010-2021, and were peer-reviewed articles reporting results that are available and written in the English language.

Exclusion criteria were secondary research other than systematic reviews and meta-analyses; interventions that did not meet the WHO definition of TH; animal studies; or studies where the participants were aged >18 years, children’s results were not reported as a separate subgroup, or there were no results reported for individuals with CP.

These criteria were assigned due to resource and funding constraints of this scoping review. Due to the recent advances in TH, it was considered that research completed before 2010 would be outdated.

Abstracts identified by the search strategy were screened by 2 investigators (MPS and TT) for eligibility using the above criteria. If it was unclear from the abstract whether the article met the eligibility criteria, the full article was retrieved for assessment.

**Figure 2:**
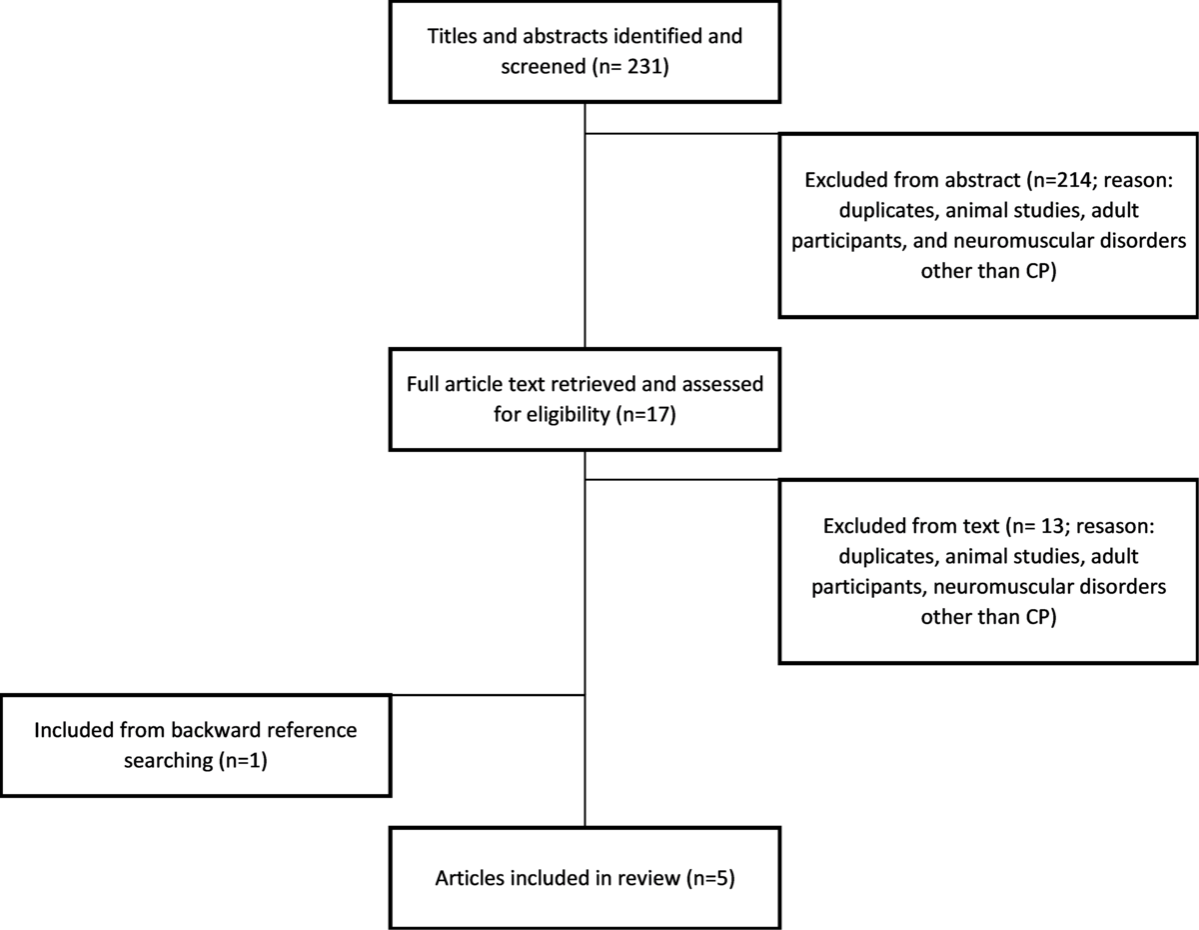
A flowchart of paper selection for inclusion in this review. CP: cerebral palsy.

The search strategy is represented in Figure 2.

## Results

### Search Results

The search was conducted on July 2, 2021. The systematic search strategy produced 231 abstracts. After reviewing the titles and abstracts, 214 articles were excluded as they were duplicates or did not meet the eligibility criteria. After reviewing the full text of 17 articles, 13 were excluded as they did not meet the eligibility criteria. From the backward reference searching of the 4 eligible articles, 1 further article was included. Therefore, 5 articles were included in this scoping review. Additionally, 2 of the included articles were systematic reviews, which included the results of 3 relevant studies each. Therefore, the results of 11 studies were included in this review [7,10,11,13-20] (Table 2). The findings of the included studies are summarized in Table 3.

**Table 2:** A summary of studies included in the scoping review.

Number	Main author	Study methodology	Study objectives
1	Preston et al [13]	Pilot study	Gaming technology on arm function
2	Hung and Fong [11]	Systematic review	Effects of telerehabilitation in occupational therapy practice
3	Ferre et al [17]	Randomized control trial	Caregiver-directed home-based intensive bimanual training
4	Golomb et al [7]	Pilot study	In-home virtual reality in telerehabilitation of adolescents with hemiplegic CP^a^
5	Reifenberg et al [16]	Case report	Pediatric game-based neurorehabilitation using TH^b^ technologies
6	Staszuk et al [15]	Pilot study	Image processing to create a “TeleReh application”
7	Tanner et al [14]	Quality improvement project	Comparing outcomes of traditional in-person therapy and TH therapy
8	Camden et al [10]	Systematic review	Telerehabilitation for children with disabilities
9	Mitchell et al [19]	Randomized control trial	Web-based intervention of a 30-minute training program
10	James et al [18]	Randomized control trial	Web-based multimodal therapy for unilateral CP
11	Chen et al [20]	Meta-analysis	Virtual reality for function

^a^CP: cerebral palsy.

^b^TH: telehealth.

**Table 3:** Findings of all included papers within the scoping review.

Number	Main author	Outcomes
1	Preston et al [13]	No outcome showing a clinically important difference in arm function improvement with gaming technology
2	Hung and Fong [11]	TH^a^ is a feasible approach for hand therapy for children, demonstrating increased motivation and hand function and reduced parental stress
3	Ferre et al [17]	Home-based therapy improved dexterity and the performance of functional goals, but not bimanual performance
4	Golomb et al [7]	Positive functional outcomes using virtual reality video game with improved hand function
5	Reifenberg et al [16]	Positive functional outcomes
6	Staszuk et al [15]	TH has the potential to assist health care services and patients in making health care more accessible, personalized, and available to all
7	Tanner et al [14]	Positive clinical utility, and COMP^b^ could accurately assess functional goals and measure change in the patient’s functional ability after receiving telerehabilitation sessions over 4 months
8	Camden et al [10]	No significant differences with web-based therapy compared to conventional face-to-face therapy, with virtual reality potentially being more effective in younger children
9	Mitchell et al [19]	Significant improvement in 6-minute walk test distance and functional strength, although no differences were noted in activity performance
10	James et al [18]	No clinically significant differences in a web-based hand therapy treatment (Move it to improve it; Mitti) when compared to usual treatment
11	Chen et al [20]	Virtual reality improves outcomes for children with CP^c^ with a strong effect size

^a^TH: telehealth.

^b^COMP: Canadian Occupational Performance Measure.

^c^CP: cerebral palsy.

### Gaming Technology

Preston et al [13] investigated gaming technology on the arm function of children with spastic CP who had recently received botulinum toxin injections. This study was a pilot and was underpowered, with 58 participants required to demonstrate a generalizable difference, but only 15 were included. The outcomes were measured with the Canadian Occupational Performance Measure (COPM), which measures patient ability, functional impairment, and satisfaction with function [14], and ABILHAND-kids, which is a parent-completed questionnaire grading the child’s ability to complete tasks. Children were randomized to usual care or the intervention, which consisted of a noncompetitive and noncollaborative computer-based game, customized for arm rehabilitation for children with CP. This intervention was provided within 1 week of the botulinum toxin injections. The results showed that the gaming system was used on average for only 7 minutes per day, despite the recommended usage being 30 minutes per day. No outcome showed a clinically important difference; however, the low recruitment numbers and the low usage of this system means that the results contribute little to the understanding of the effectiveness of computer-based gaming systems on remote rehabilitation [13].

Hung and Fong [11] published a systematic review in 2019 on the effects of TH in occupational therapy practice. The review included 15 studies, 3 of which included children with CP [7,16,17]. These studies investigated hand function training through teleconsultation and telemonitoring [16,17] and a virtual reality video game home program [7]. Ferre et al [17] trained caregivers to take standardized assessments with the patients; Golomb et al [7] and Reifenberg et al [16] used occupational therapist–measured outcomes remotely and in clinic. Together, the studies demonstrated that TH was a feasible approach for hand therapy for children with CP and demonstrated increased motivation [16] and hand function [7,16] and reduced parental stress [16].

### Image Processing

Staszuk et al [15] investigated a system of TH as it applied to children with CP while also including adults with stroke. The proposed system involved (1) a central database where all patient information, medical tests, results, and images were stored; (2) a network of rehabilitation centers, laboratories, and hospitals that were involved in patients’ care; (3) a web service that connected the stored data from the database with all the health care professionals involved, through a secure internet connection; and (4) a computer or other electronic device (tablet, smartwatch, or smartphone) in the child’s home that could send relevant data and information to the central database, thus creating a health record for each patient that was concise enough to allow health care professionals to monitor their health and recovery sufficiently [15].

The TH process used cameras to look at the patient’s hand movements and a “TeleReh” application [15] where a predefined standard of movements was set for the child to practice and perfect. Each time they managed to complete the movement, the software detected it and moved onto the next step of the rehabilitation process. These steps formed a training program and used rehabilitation techniques remotely, without the need of a health care professional to be physically present, although they could be present over the web to watch and assist the rehabilitation process [15].

The main technology behind this system was image processing. The proposed stages included (1) processing the video into picture frames and (2) using local thresholding to identify homogenous areas, which led to (3) hand detection based on hand texture, colors, and shapes. This research concluded that TH has potential to assist health care services and children in making health care more accessible, personalized, and available to all [15].

### Teleconsultations and Virtual Therapies

Tanner et al [14] compared the outcomes of conventional in-person therapy and TH therapy as a quality improvement project following a forced change to TH during the COVID-19 pandemic. In their study, they used COPM to set personalized goals for children by identifying the individual challenges they encountered and measured the outcomes of the telerehabilitation process. The TH therapy was delivered through videoconferencing, and all therapists had previously been trained specifically in pediatric patients. Tanner et al [14] conducted 3 cycles in which they (1) identified what measure to use, (2) assessed if it was a feasible measure to use, and (3) looked into the therapists’ perceptions of its use [14]. The results were promising, showing that there was a positive clinical utility and COPM could accurately assess functional goals and measure change in the child’s functional ability after receiving telerehabilitation sessions over a period of 4 months [14].

A further systematic review was conducted in 2020 by Camden et al [10], investigating telerehabilitation for children with disabilities; 3 included studies involved participants who were children with CP and were randomized controlled trials of web-based games and a meta-analysis of virtual reality training [18-20].

A paper by James et al [18] was included in this systematic review, which showed that there were no clinically significant differences in a web-based hand therapy treatment (Move it to improve it; Mitti) when compared to usual treatment, indicating that this program is a viable therapy tool. The outcomes measured were primarily focused on hand function, including the Assessment of Motor and Process Skills and COPM [18].

This systematic review also included the only study that investigated a full-body intervention. All other included studies investigated interventions for the hand only. Mitchell et al [19] conducted a randomized control trial on an individualized web-based intervention, which allowed participants to undertake a daily program of 30 minutes of training. This study included a mix of repetitive body weight exercises and interactive games. This intervention indicated significant improvement in the 6-minute walk test and functional strength, although no differences were noted in activity performance [19].

Finally, Chen et al [20] meta-analyzed 14 articles, which indicated that virtual reality improved outcomes for children with CP with a strong effect size. However, it was noted that the poor quality of the studies meant that further high-quality research was required to reach a firm recommendation. A subgroup analysis indicated that virtual reality may be more effective for younger children [20].

## Discussion

The aim of this scoping review was to analyze and summarize the existing evidence for TH interventions for the treatment of children with CP and identify any areas requiring further research.

### Principal Findings

We have found the existing evidence for TH interventions for the treatment of children with CP to be scarce and of poor quality, primarily comprising of underpowered pilot studies. There was reasonable consistency in the domains of outcomes measured, with most studies investigating activity, participation, and patient and parent satisfaction. However, most studies measured these domains using different tools; therefore, result comparison across studies was limited. Most studies did not include an adequate description of participants and often lacked CP neurological type or GMFCS level; therefore, the results were difficult to interpret for clinical application.

The scope of the research into TH was often broad, whereas patient recruitment was limited, leading to underpowered results. Difficulties in assessing the efficiency and efficacy of these devices and services, as well as the broad definitions, make it difficult to directly compare their outcomes and combine results.

However, in the aggregate, the results of TH interventions for the treatment of children with CP were positive, indicating either comparable results with children receiving usual face-to-face care or in some domains, the results were improved. Notably, there is some evidence that TH may increase participant motivation with therapy, reduce caregiver stress, increase functional abilities, and may improve the accessibility of health services. These findings are supported by previous reviews, which have found that assistive technologies for children with physical disabilities increase motivation and reduce caregiver burden [21].

The only study that did not demonstrate any promising results was the study by Preston et al [13], who investigated the ability of a customized computer-based gaming system to improve the arm function of children with spastic CP. However, the children were not compliant with the treatment, averaging only 7 minutes of game play per day, which was significantly lower than the recommended 30 minutes. This finding contrasts with other studies included in this review, which tested home gaming systems; Golomb et al [7], Reifenberg et al [16], and Chen et al [20] reported positive functional outcomes and noted minimal difficulties with compliance. However, it should be noted that the paper by Reifenberg et al[16] is a single case report. Given the functional improvements noted by the other 3 studies, it is possible that the game proposed by Preston et al [13] was less compelling or that a nonrepresentative sample was recruited for the study leading to type II error. This finding emphasizes the uncertainties in interpreting results presented by an underpowered study.

The results for TH without a computer-based or web-based game aspect also showed potential for functional improvements in children with unilateral spastic CP. Ferre et al [17] taught caregivers via TH, child-friendly, and home-based activities designed to improve bimanual hand use, including board games, clay modeling, and page turning. This approach encouraged family-centered care, placing caregivers rather than therapists at the center of the child’s care and allowing intense therapy without the additional caregiver burden of hospital travel and appointments conflicting with work schedules. However, it should be noted that there was high attrition from the study, with 40% of participants not completing the intervention. Further research should be conducted to established if this result was due to a flaw in study or intervention design. Importantly, this study also showed no significant differences between the therapist-measured baseline and caregiver-measured baseline, indicating that the Assisting Hand Assessment and Box and Blocks Test can be appropriately measured by caregivers remotely at baseline [17].

Although these results are promising for TH as an intervention for the hand in children with CP, it should be noted that there is insufficient evidence on TH interventions for other areas of the body, including the lower limbs or body posture. Mitchell et al [19] conducted the only study included in this scoping review that investigated a full-body intervention. This intervention included body weight exercises and interactive games that participants undertook at home under the guidance and supervision of remote therapists. Although the results of this study were promising, showing excellent adherence to the program and improved functional measures, they did not translate into significant differences in activity-based outcome measures [19]. However, this finding is not uncommon for this patient group, and the ceiling effects of training can also be seen in face-to-face therapy [22]. Therefore, the findings may be considered as showing promise for TH as a full-body intervention for children with CP [19].

Importantly, we have not identified any investigation on pain management through TH. This TH type is particularly important as more than half (67.1%) of children with CP report acute pain [23], and this pain appears to have increased through the lack of intervention during the COVID-19 pandemic [24].

### Future Directions

Of critical and immediate importance to improving the existing evidence base for TH for children with CP is the development of studies with good methodological quality and a sufficient cohort to draw appropriately powered conclusions. The majority of the included studies in this scoping review were case series, which notably complicates the interpretation of results, as where results conflict, it is difficult to assess whether this conflict is due to intervention differences, problems with study methodology, or type II error.

There is some evidence that the requirements of TH vary depending on developmental stage [25] and GMFCS level [26]. It is likely that different neurological types of CP (dyskinetic, dystonic, or ataxic) will have different requirements of TH and require adaptions to accommodate different movements. For example, mathematical models have been developed to improve the layout of touchscreen communication devices for children with dyskinetic CP with different motor requirements, with good results [27]. However, this adaptation has not been adequately investigated to suggest what forms of TH are effective for different children with CP, partially due to small study sizes but also due to the poor definition of participants in studies. Therefore, future work would benefit from clearly defining the participant inclusion criteria, descriptive statistics, and subgroup analysis stratification by GMFCS level, age, and CP neurological type. This definition will increase the understanding of the effectiveness of TH for different groups of children with CP and allow greater potential for meta-analysis.

There is also limited evidence of the barriers to TH implementation, particularly in the longer term. Most of the existing evidence is anecdotal or a short description of the reasons for attrition from studies. A larger qualitative study, investigating the reasons for dropout from TH studies or noncompliance with TH necessarily implemented during the COVID-19 pandemic, would provide a much greater understanding of the barriers to TH uptake.

Future work would benefit from focusing on outcomes that are important to children with CP. Considering outcomes, such as pain and spasticity, that have been shown to have deteriorated through the lack of in-person treatment during the COVID-19 pandemic would also be useful. Parent-reported outcome measures were commonly gathered across TH studies, allowing the collection of data remotely. The most commonly used outcome measure was the COPM. However, it should be noted that the COPM has only been validated for children with spastic CP. Further work would be required to validate its appropriateness for children with ataxic and dyskinetic CP [28]. The validation of the COPM in larger patient groups will likely be beneficial to this area of research. However, as most therapists agree, this validation should be applicable within a reasonable length of time, and functional goals should be identified [14].

Finally, more evidence is required to support the use of TH in the treatment of the lower limb or postural control for children with CP. The existing evidence is strongly focused on the upper limb. It is possible that retrospective studies of routinely collected clinical outcomes during the COVID-19 pandemic may provide some valuable insight into this area of TH.

### Limitations

This scoping review included the results of 11 studies—a small number despite the broad eligibility criteria, which included any therapy, study aims, and outcomes measured. There are limited trials that have been conducted in this area despite the drastic increase in TH consultations reported through the COVID-19 pandemic. Retrospective research using routinely collected outcome measures in this patient group gathered throughout the COVID-19 pandemic may add important findings and context for the potential benefits of ongoing TH consultations. We would recommend the publication of any data gathered by health care professionals on their changing interventions in this time. As COVID-19 has affected the need for more TH, this subject is likely to expand exponentially in the future and more reviews similar to this paper will be needed for further assessment.

Second, the substantial heterogeneity of participants made comparison of outcomes between studies challenging. To reduce the heterogeneity, classifications systems such as the GMFCS have been used. However, the GMFCS levels or CP neurological type were not consistently reported; therefore, it is unclear if the results are generalizable to the population or comparable to other forms of TH. This result has limited our drawing of clinical recommendations on TH for the treatment of children with CP, but it has helped identify limitations in the literature as a whole and addressed a key issue that needs careful consideration in future primary research investigating CP treatment.

Third, due to the heterogeneity of participants and the inconsistency in outcome measures used, it was not possible to directly compare the results of studies through meta-analysis. Therefore, we were unable to directly compare the quantitative results of the studies and examine statistically and clinically significant differences across smaller studies, which has affected our efforts to chart data and directly visualize results quantitatively. As the technological advancements increase and the quality of the research improves, we hope that in the future, there will be more quantitative data to enable direct analysis and comparison, which will inform clinical recommendations for TH.

Finally, due to time and resource constraints, we were unable to perform a formal critical appraisal of the included studies, which may mean that the results are unduly influenced by research with poor methodology. We have tried to identify notable areas of concern with research methodology within the narrative review, as a less formal critical review. However, future work in this area should consider a formal critical review.

### Conclusions

This scoping review provided for the first time an up-to-date account of the current evidence on TH for children with CP. The evidence has been shown to be lacking due to poor study design or underpowered results; therefore, clinical recommendations were not possible. However, this scoping review has shown that TH has demonstrated its potential to improve hand function while making health care services more accessible. TH interventions have shown similar or improved results compared to face-to-face treatment. To understand precisely how this technology will benefit children with CP, further research is required, with a focus on the lower limb, postural control, and quite importantly, pain—a substantial barrier to interventions being accepted by children. Further work to identify barriers to TH implementation is also required.

## Appendix

Multimedia Appendix 1 Completed PRISMA-ScR (Preferred Reporting Items for Systematic Reviews and Meta-Analyses Extension for Scoping Reviews) checklist.

## Abbreviations

BG: basal ganglia
COPM: Canadian Occupation Performance Measure
CP: cerebral palsy
GMFCS: Gross Motor Function Classification System
PRISMA-ScR: Preferred Reporting Items for Systematic Reviews and Meta-Analyses Extension for Scoping Reviews
TH: telehealth
WHO: World Health Organization

## References

[1] Bax M, Goldstein M, Rosenbaum P, Leviton A, Paneth N, Dan B, Jacobsson B, Damiano D, Executive Committee for the Definition of Cerebral Palsy (2005). Proposed definition and classification of cerebral palsy, April 2005. Dev Med Child Neurol.

[2] Cerebral palsy. BMJ Best Practice.

[3] Rethlefsen SA, Ryan DD, Kay RM (2010). Classification systems in cerebral palsy. Orthop Clin North Am.

[4] Morris C, Bartlett D (2004). Gross motor function classification system: impact and utility. Dev Med Child Neurol.

[5] (2012). Spasticity in under 19s: management clinical guideline. National Institute for Health Care Excellence.

[6] (2010). Telemedicine: opportunities and developments in member states: report on the second global survey on eHealth. World Health Organization.

[7] Golomb MR, McDonald BC, Warden SJ, Yonkman J, Saykin AJ, Shirley B, Huber M, Rabin B, Abdelbaky M, Nwosu ME, Barkat-Masih M, Burdea GC (2010). In-home virtual reality videogame telerehabilitation in adolescents with hemiplegic cerebral palsy. Arch Phys Med Rehabil.

[8] Edirippulige S, Reyno J, Armfield NR, Bambling M, Lloyd O, McNevin E (2016). Availability, spatial accessibility, utilisation and the role of telehealth for multi-disciplinary paediatric cerebral palsy services in Queensland. J Telemed Telecare.

[9] Doraiswamy S, Abraham A, Mamtani R, Cheema S (2020). Use of telehealth during the COVID-19 pandemic: scoping review. J Med Internet Res.

[10] Camden C, Pratte G, Fallon F, Couture M, Berbari J, Tousignant M (2020). Diversity of practices in telerehabilitation for children with disabilities and effective intervention characteristics: results from a systematic review. Disabil Rehabil.

[11] Hung Kn G, Fong KN (2019). Effects of telerehabilitation in occupational therapy practice: a systematic review. Hong Kong J Occup Ther.

[12] Tricco Andrea C, Lillie Erin, Zarin Wasifa, O'Brien Kelly K, Colquhoun Heather, Levac Danielle, Moher David, Peters Micah D J, Horsley Tanya, Weeks Laura, Hempel Susanne, Akl Elie A, Chang Christine, McGowan Jessie, Stewart Lesley, Hartling Lisa, Aldcroft Adrian, Wilson Michael G, Garritty Chantelle, Lewin Simon, Godfrey Christina M, Macdonald Marilyn T, Langlois Etienne V, Soares-Weiser Karla, Moriarty Jo, Clifford Tammy, Tunçalp Özge, Straus Sharon E (2018). PRISMA Extension for Scoping Reviews (PRISMA-ScR): checklist and explanation. Ann Intern Med.

[13] Preston N, Weightman A, Gallagher J, Levesley M, Mon-Williams M, Clarke M, O'Connor RJ (2016). A pilot single-blind multicentre randomized controlled trial to evaluate the potential benefits of computer-assisted arm rehabilitation gaming technology on the arm function of children with spastic cerebral palsy. Clin Rehabil.

[14] Tanner LR, Grinde K, McCormick C (2021). The Canadian Occupational Performance Measure: a feasible multidisciplinary outcome measure for pediatric telerehabilitation. Int J Telerehabil.

[15] Staszuk A, Wiatrak B, Tadeusiewicz R, Karuga-Kuźniewska E, Rybak Z (2016). Telerehabilitation approach for patients with hand impairment. Acta Bioeng Biomech.

[16] Reifenberg G, Gabrosek G, Tanner K, Harpster K, Proffitt R, Persch A (2017). Feasibility of pediatric game-based neurorehabilitation using telehealth technologies: a case report. Am J Occup Ther.

[17] Ferre Claudio L, Brandão Marina, Surana Bhavini, Dew Ashley P, Moreau Noelle G, Gordon Andrew M (2017). Caregiver-directed home-based intensive bimanual training in young children with unilateral spastic cerebral palsy: a randomized trial. Dev Med Child Neurol.

[18] James S, Ziviani J, Ware RS, Boyd RN (2015). Randomized controlled trial of web-based multimodal therapy for unilateral cerebral palsy to improve occupational performance. Dev Med Child Neurol.

[19] Mitchell LE, Ziviani J, Boyd RN (2016). A randomized controlled trial of web-based training to increase activity in children with cerebral palsy. Dev Med Child Neurol.

[20] Chen Y, Lee S, Howard AM (2014). Effect of virtual reality on upper extremity function in children with cerebral palsy: a meta-analysis. Pediatr Phys Ther.

[21] Nicolson A, Moir L, Millsteed J (2012). Impact of assistive technology on family caregivers of children with physical disabilities: a systematic review. Disabil Rehabil Assist Technol.

[22] Smits DW, Gorter JW, van Schie PE, Dallmeijer AJ, Ketelaar M, PERRIN+ study group (2014). How do changes in motor capacity, motor capability, and motor performance relate in children and adolescents with cerebral palsy?. Arch Phys Med Rehabil.

[23] Ostojic K, Paget S, Kyriagis M, Morrow A (2020). Acute and chronic pain in children and adolescents with cerebral palsy: prevalence, interference, and management. Arch Phys Med Rehabil.

[24] Karatekin BD, İcagasioglu A, Sahin SN, Kacar G, Bayram F (2021). How did the lockdown imposed due to COVID-19 affect patients with cerebral palsy?. Pediatr Phys Ther.

[25] Huang IC, Sugden D, Beveridge S (2009). Assistive devices and cerebral palsy: the use of assistive devices at school by children with cerebral palsy. Child Care Health Dev.

[26] Ostensjø Sigrid, Carlberg EB, Vøllestad Nina K (2005). The use and impact of assistive devices and other environmental modifications on everyday activities and care in young children with cerebral palsy. Disabil Rehabil.

[27] Bertucco M, Sanger TD (2018). A model to estimate the optimal layout for assistive communication touchscreen devices in children with dyskinetic cerebral palsy. IEEE Trans Neural Syst Rehabil Eng.

[28] Cusick A, Lannin NA, Lowe K (2007). Adapting the Canadian Occupational Performance Measure for use in a paediatric clinical trial. Disabil Rehabil.

